# Material and social deprivation associated with public health actual causes of death among older people in Europe: longitudinal and multilevel results from the Survey of Health, Ageing and Retirement in Europe (SHARE)

**DOI:** 10.3389/fpubh.2024.1469203

**Published:** 2024-10-29

**Authors:** Matthias Hans Belau

**Affiliations:** University Medical Centre Hamburg-Eppendorf, Institute of Medical Biometry and Epidemiology, Hamburg, Germany

**Keywords:** social class, mortality, neoplasm, heart attack, stroke, Europe

## Abstract

**Background:**

Adverse socioeconomic conditions at the individual and regional levels are associated with an increased risk of mortality. However, few studies have examined this relationship using multilevel analysis and, if so, only within a single country. This study aimed to examine this relationship using data from several European countries.

**Methods:**

Individual-level data were obtained from Waves 5 to 9 of the Survey of Health, Ageing and Retirement in Europe, while regional-level data were obtained from the Luxembourg Income Study Database. Cox regression analysis with gamma-shared frailty and a random intercept for country of residence was used to examine the association between individual mortality from all causes, cancer, heart attack, and stroke and measures of socioeconomic deprivation at the individual level, including material and social deprivation indices, and at the area level, including the Gini index.

**Results:**

The risk of mortality from all causes was increased for respondents with material deprivation (hazard ratio (HR) = 1.77, 95% CI = [1.60, 1.96]) and social deprivation (HR = 7.63, 95% CI = [6.42, 9.07]) compared with those without. A similar association was observed between individual deprivation and the risk of mortality from cancer, heart attack, or stroke. Regional deprivation had a modest contextual effect on the individual risk of death from all causes and cancer. However, when individual-level deprivation was included in the models, no contextual effects were found.

**Conclusion:**

The results indicate that individual socioeconomic conditions significantly predict causes of death in older European adults, with those with material deprivation and social deprivation having a higher risk of death from all causes, including cancer, heart attack, and stroke, while the Gini index has a minimal effect, although the Gini index reflects regional disparities across Europe.

## Introduction

Life expectancy has increased markedly in numerous European countries over the past few decades (1). This favorable trend can be attributed to advances in the diagnosis, treatment, and prevention of common diseases that are major causes of death (2–4). Nevertheless, despite these improvements, cardiovascular disease and cancer, with a median onset age of 65–70 years (5–8), remain the leading causes of death in Europe (9, 10). They also have a significant economic impact (11).

It has been proposed that adverse socioeconomic conditions at the individual and regional levels contribute to the development of cardiovascular disease, including ischemic heart disease [i.e., heart attack (12–14)] and cerebrovascular disease [i.e., stroke (15, 16)]. Furthermore, studies have demonstrated associations between socioeconomic indicators and certain cancers (17–19).

The concept of individual socioeconomic status is constituted by a complex network of interrelated factors that are inextricably linked to an individual’s material and social context. Such factors include, but are not limited to, educational attainment, income, and occupation (20). Nevertheless, income-based measures may prove to be particularly inadequate proxies for material conditions in older individuals, for example, when they are retired (21). In addition to the potential for financial losses, the transition to retirement is frequently accompanied by alterations in an individual’s social networks and participation in shared activities (22, 23). These changes have the potential to influence an individual’s health (24, 25) and quality of life (26). In contrast, regional socioeconomic measures, as exemplified by single measures or multidimensional deprivation indices, focus on material and social indicators at the area level (27, 28).

The extant literature examines the relationship between individual or regional socioeconomic measures and the underlying causes of death within a country. However, few studies have considered both individual and regional levels of analysis simultaneously using a multilevel approach, which constrains the inferences that can be drawn from existing studies. Moreover, there are notable socioeconomic and health disparities between European countries, as evidenced by the findings of numerous studies (29, 30), which further constrains the external validity of these studies.

The objective of this study was to examine the association between socioeconomic deprivation and the actual causes of death among older people in the European Region, with consideration of area-level information in addition to individual-level information. The hypothesis was that elevated mortality risk is associated with higher levels of socioeconomic deprivation at both the individual and area levels.

## Materials and methods

### Study population and procedure

The data from Waves 5 to 9 of the Survey of Health, Ageing and Retirement in Europe (SHARE) (31) were subjected to analysis. The SHARE population included individuals aged 50 and older and their spouses and partners, regardless of age, from 14 European countries and Israel who were permanent residents of each country. Individual data were available at the major socioeconomic region level (NUTS 1) for respondents in Belgium, Germany, Israel, Italy, Slovenia, Spain (only the Autonomous Community of Este), and Sweden, and at the country level (NUTS 0) for respondents in Austria, the Czech Republic, Denmark, Estonia, France, Luxembourg, the Netherlands, Spain (excluding the Autonomous Community of Este), and Switzerland. Wave 5, which was administered in 2013, encompassed computer-assisted personal interview data for 66,038 individuals. This data included information on individual-level socioeconomic deprivation, specifically on material and social deprivation. Subsequent follow-up was conducted approximately every 2 years for a period exceeding 8 years (Waves 6 to 9). This included information on vital status and disease burden. A total of 9,509 respondents lacked information on material and social deprivation and thus were excluded from the analysis. In addition, 968 respondents were less than 50 years of age and were also excluded, as the deprivation index utilized (see below) is validated for the 50-year-old and older population (32). The final analytical sample consisted of *N* = 55,561 individuals for the material and social deprivation and cause of death analyses.

### Measures

#### Ascertainment of vital status

Information on vital status was obtained from the status of their participation in the follow-up period. If a participant completed the survey in the corresponding Waves 6 to 9, their vital status was designated as “alive.” When information was available on individuals who died during the follow-up period, the cause of death was documented by conducting an “end-of-life interview” with close relatives of the deceased respondent. The outcomes of interest in this study were all-cause death and death due to public health causes, including cancer, stroke, and heart attack. The reported time of death (month/year) was defined as the endpoint of the observation period.

#### Area-level variable

The Gini index was employed to evaluate regional deprivation for each country and for the corresponding SHARE Wave 5 to 9 years. This approach is consistent with that employed in other studies that have examined area-level socioeconomic deprivation in European countries (33, 34). The Gini index is a measure of the extent to which the distribution of income or consumption among individuals or households within an economy deviates from a perfectly equal distribution. The Gini index is defined on a scale of 0 to 100, with higher values indicating greater levels of inequality. The data were obtained from the Luxembourg Income Study (LIS) Database (35), which contains harmonized microdata collected from approximately 50 countries across Europe, North America, Latin America, Africa, Asia, and Australasia, spanning five decades. The Gini indices utilized in this analysis were constructed with weighted data on household income from population surveys conducted during the SHARE Waves 5 to 9 (i.e., in the years 2013, 2015, 2017, 2019, and 2021). It is important to note that the Gini index does not indicate the absolute level of income of the population. This implies that an area or country with a very low absolute income can have an equal income distribution and still have a very poor population. Consequently, an adjustment was made for the relative poverty rate, as detailed below.

#### Individual-level variables

Material deprivation was operationalized at the individual level by aggregating 11 items related to the affordability of basic needs and the prevalence of financial difficulties (36). The items are listed in Table 1 and described in detail elsewhere (37). They address various aspects of economic circumstances. These include the ability to afford meat or fruit more than three times per week, the affordability of specific items such as groceries and vacations, the necessity to limit expenses in items such as footwear or heating to maintain affordable living costs, and the inability to access medical care due to financial constraints. The material deprivation index (MDI) aggregates binary indicators on whether an individual is deprived of a specific item, employing so-called “hedonic weights” derived from a multiple regression of all items on a single life satisfaction measure (32, 36). The final MDI is defined on a scale from 0 to 1, with higher values indicating a greater degree of deprivation.

**Table 1:** Material and social deprivation items.

Material deprivation index	Social deprivation index
Affordability of:	1. Less than one room per person in household
1. Meat, fish, or chicken	2. Poor reading or writing skills
2. Fruits, or vegetables	3. Poor computer skills or never used a computer
3. Regular grocery shopping	4. Not feeling part of the local area
4. A week-long vacation once a year	5. Vandalism is a problem in the local area
5. Unexpected expense	6. Local area is not clean
Keeping living costs down by:	7. No helpful people in the local area
6. Wearing worn-out clothes	Having difficulties to access:
7. Wearing worn-out shoes	8. Bank
8. Reducing heating	9. Grocery shop
9. Not replacing glasses	10. Pharmacy
10. Postponing dentist	11. Waiting too long to see a doctor
11. Postponing doctor	12. Not attending any course
	13. Not taking part in any organization
	14. Not trusting people
	15. Feeling left out of things

Social deprivation was operationalized at the individual level by aggregating 15 items related to participation in everyday life, social activities, and the quality of the neighborhood. The items are listed in Table 1 and described in detail in another source (38). They comprise social isolation and lack of social support, as well as normative integration. The social deprivation index (SDI) also aggregates binary indicators on whether an individual is deprived of a specific item, employing the “hedonic weights” derived from a multiple regression of all items on a single life satisfaction measure (32, 36, 38). The final SDI is defined on a scale from 0 to 1, with higher values indicating a greater degree of deprivation.

#### Covariates

Area-level covariates encompass data on the percentage of the migrant population and the relative poverty rate. The data were obtained from the Luxembourg Income Study (LIS) Database (35). The relative poverty rate represents the proportion of households whose incomes fall below the median equivalized household disposable income in the total population and corresponding year. Covariates at the individual level encompass data on age, sex (female, male), education (low, medium, high), migration background (absent, one-sided, two-sided), and disease burden. A two-sided migration background is defined as the case of individuals with both parents who are citizens of a foreign country or who were born in a country other than the one in which they currently reside. In contrast, a one-sided migration background is defined as the case of individuals with only one parent who is a citizen of a foreign country or who was born in a country other than the country in which the respondent currently resides. The disease burden was quantified through a modified version of the Charlson comorbidity index (39, 40), wherein the following diseases (ever diagnosed) were assigned weighted scores: a score of two was assigned to conditions including heart attack or congestive heart failure, Alzheimer’s disease or dementia, cancer, and stroke; a score of one was assigned to chronic pulmonary disease, rheumatologic disease, and diabetes. Subsequently, the index was calculated by summing the weighted scores of diseases self-reported by the individuals, with higher scores indicating a greater burden of disease.

### Statistical analysis

Descriptive statistics were employed to ascertain respondents’ and country-specific characteristics. To illustrate the regional distribution of the Gini index, a quintile-based choropleth map was created. Furthermore, Kaplan–Meier curves were created for all-cause mortality among subgroups of MDI, categorized into unexposed (due to a spike at zero, as illustrated below) and tertiles of MDI greater than zero (low, medium, high) and SDI, categorized into quartiles (low, medium-low, medium-high, high). The association between socioeconomic deprivation and individual mortality was evaluated using a Cox regression model with gamma shared frailty and a random intercept for country of residence. The independent variables considered in the regression models for all-cause mortality (model 1), cancer mortality (model 2), heart attack mortality (model 3), and stroke mortality (model 4) were the Gini index (basic model) as well as MDI and SDI (full model). These variables were analyzed as continuous variables. To account for demographic and health differences, the regression models (basic and full models) were adjusted for the aforementioned area-level and individual-level covariates. Explained variation (*R*^2^) for basic and full survival models was measured according to Royston (41). As the exposure variable MDI comprises positive continuous values with a spike at zero (SAZ), a number of different linear methods were evaluated for model selection. These included (i) modeling the SAZ variable continuously and untransformed, (ii) modeling it using the fractional polynomial (FP) approach, (iii) modeling it continuously and untransformed, including a binary indicator for SAZ, and (iv) modeling it using the FP method, including a binary indicator for the SAZ, as proposed by Lorenz and colleagues (42). The results of the model selection for each outcome of interest can be found in Supplementary material S1. The association between individual socioeconomic deprivation and individual mortality stratified by quintiles of the Gini index was also examined. The stratified models were adjusted for the aforementioned area-level and individual-level covariates to account for potential confounding factors. The proportional hazards assumption was evaluated through a graphical examination of the Schoenfeld residuals, and no serious violation of the assumption was observed. Associations were presented as hazard ratios (HR) and 95% confidence intervals (CI). All analyses were performed with STATA/MP 18.

## Results

Table 2 provides a summary of the characteristics of the study population for SHARE Waves 5 to 9. The sample was composed of slightly more women (55.8%) than men (44.2%), and the majority of the participants did not have a migration background (80.9%). The mean age of the participants was 69.4 years. One-fourth of the participants reported high levels of education.

**Table 2:** Characteristics of the SHARE study population^†^ by Wave.

	Wave 5 2013	Wave 6 2015	Wave 7 2017	Wave 8 2019	Wave 9 2021
Total	55,561	47,888	41,989	33,881	26,857
Vital status					
Alive	55,561	45,881 (95.8)	40,193 (95.7)	31,921 (94.2)	25,381 (94.5)
Deceased, all-cause	–	2,007 (4.2)	1,796 (4.3)	1,960 (5.8)	1,476 (5.5)
Cancer^ƒ^	–	597 (29.7)	455 (25.3)	450 (23.0)	219 (14.8)
Heart attack^ƒ^	–	233 (11.6)	211 (11.7)	187 (9.5)	79 (5.4)
Stroke^ƒ^	–	177 (8.8)	146 (8.1)	157 (8.0)	93 (6.3)
Individual characteristics					
Age, years	66.3 ± 9.7	68.3 ± 9.7	70.0 ± 9.5	71.9 ± 9.2	73.3 ± 8.7
Sex					
Female	30,710 (55.3)	26,421 (55.2)	23,428 (55.8)	19,102 (56.4)	15,408 (57.4)
Male	24,851 (44.7)	21,467 (44.8)	18,561 (44.2)	14,779 (43.6)	11,449 (42.6)
Education					
High	13,049 (23.5)	11,349 (23.7)	10,197 (24.3)	8,620 (25.4)	7,130 (26.6)
Medium	21,004 (37.8)	18,070 (37.7)	15,995 (38.1)	13,251 (39.1)	10,723 (39.9)
Low	21,508 (38.7)	18,469 (38.6)	15,797 (37.6)	12,010 (35.5)	9,004 (33.5)
Migration background					
Two-sided	7,770 (14.0)	6,506 (13.6)	5,539 (13.2)	4,143 (12.2)	3,107 (11.6)
One-sided	3,241 (5.8)	2,769 (5.8)	2,449 (5.8)	2,065 (6.1)	1,663 (6.2)
Absent	44,550 (80.2)	38,613 (80.6)	34,001 (81.0)	27,673 (81.7)	22,087 (82.2)
Charlson comorbidity index	0.8 ± 1.0	0.7 ± 1.0	0.7 ± 1.0	0.6 ± 1.0	0.9 ± 1.1
Country of residence					
Austria	3,988 (7.2)	3,518 (7.4)	3,120 (7.4)	2,540 (7.5)	2,177 (8.1)
Belgium	4,924 (8.9)	4,271 (8.9)	3,740 (8.9)	3,005 (8.9)	2,635 (9.8)
Czech Republic	4,005 (7.2)	3,573 (7.5)	3,104 (7.4)	2,424 (7.1)	1,799 (6.7)
Denmark	3,595 (6.5)	3,229 (6.7)	2,868 (6.8)	2,388 (7.1)	1,801 (6.7)
Estonia	5,135 (9.2)	4,857 (10.1)	4,382 (10.4)	3,741 (11.0)	3,083 (11.5)
France	4,067 (7.3)	3,338 (7.0)	2,834 (6.7)	2,294 (6.8)	1,753 (6.5)
Germany	4,968 (9.0)	3,975 (8.3)	3,421 (8.2)	2,863 (8.5)	2,282 (8.5)
Israel	1,350 (2.4)	1,224 (2.6)	1,061 (2.5)	630 (1.9)	323 (1.2)
Italy	4,060 (7.3)	3,680 (7.7)	3,327 (7.9)	2,797 (8.3)	2,521 (9.4)
Luxembourg	1,444 (2.6)	1,136 (2.4)	925 (2.2)	725 (2.1)	540 (2.0)
Netherlands	3,372 (6.1)	2,021 (4.2)	2,000 (4.8)	1,972 (5.8)	1,560 (5.8)
Slovenia	2,590 (4.7)	2,361 (4.9)	2,120 (5.1)	1,881 (5.5)	1,638 (6.1)
Spain	5,411 (9.7)	4,864 (10.2)	4,009 (9.6)	2,384 (7.0)	1,385 (5.2)
Sweden	3,890 (7.0)	3,360 (7.0)	2,923 (7.0)	2,437 (7.2)	1,936 (7.2)
Switzerland	2,762 (4.9)	2,481 (5.1)	2,155 (5.1)	1,800 (5.3)	1,424 (5.3)

Data are shown as *n* (%) or mean ± standard deviation; n, quantity; %, proportion.

† Sample is restricted to SHARE respondents aged 50 years and older; ^ƒ^ all-cause death is denominator.

**Figure 1:**
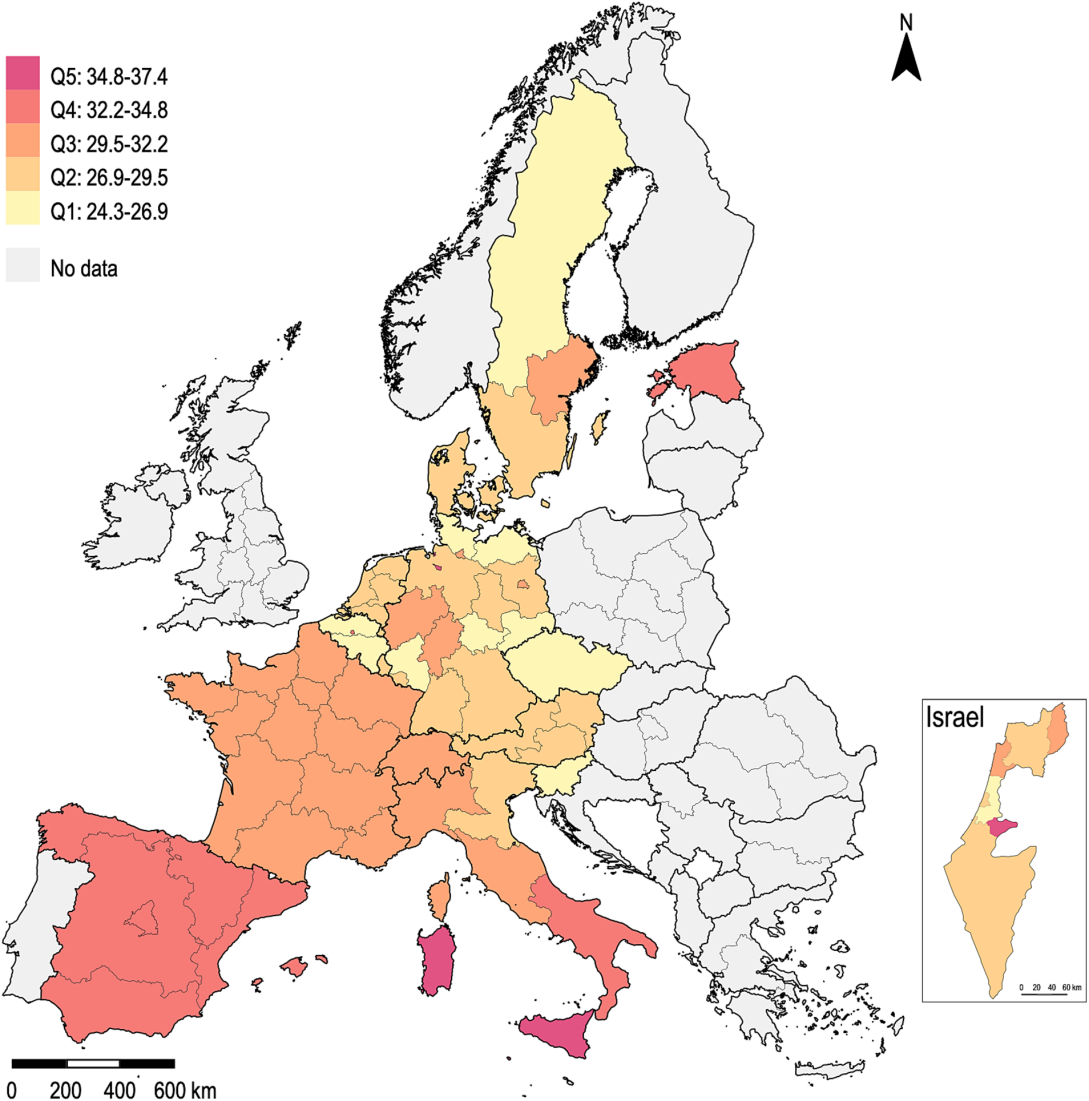
Distribution of the Gini index, the arithmetic mean of the years 2013 to 2021.

The country’s Gini index exhibited a range from a minimum of 24.9 in the Czech Republic to a maximum of 40.7 in Israel, with a mean value of 29.4 and a standard deviation of 2.9 across all countries. Figure 1 depicts the regional disparities observed in the distribution of the Gini index quintiles. A more detailed illustration of the country’s Gini index across SHARE Waves 5 to 9 can be found in Supplementary material S2. The distribution of the relative poverty rate and the percentage of the migrant population can be found in Supplementary materials S3, S4, respectively.

The mean value ± standard deviation for material deprivation index (MDI) was 0.13 ± 0.19 (min, max: 0.00, 0.99) across all countries, while that for social deprivation index (SDI) was 0.17 ± 0.14 (min, max: 0.00, 0.92). The respondents from Denmark exhibited the lowest mean values for both MDI = 0.04 and SDI = 0.09, while those from Estonia (MDI = 0.31, SDI = 0.22) and Italy (MDI = 0.21, SDI = 0.24) demonstrated the highest.

**Figure 2:**
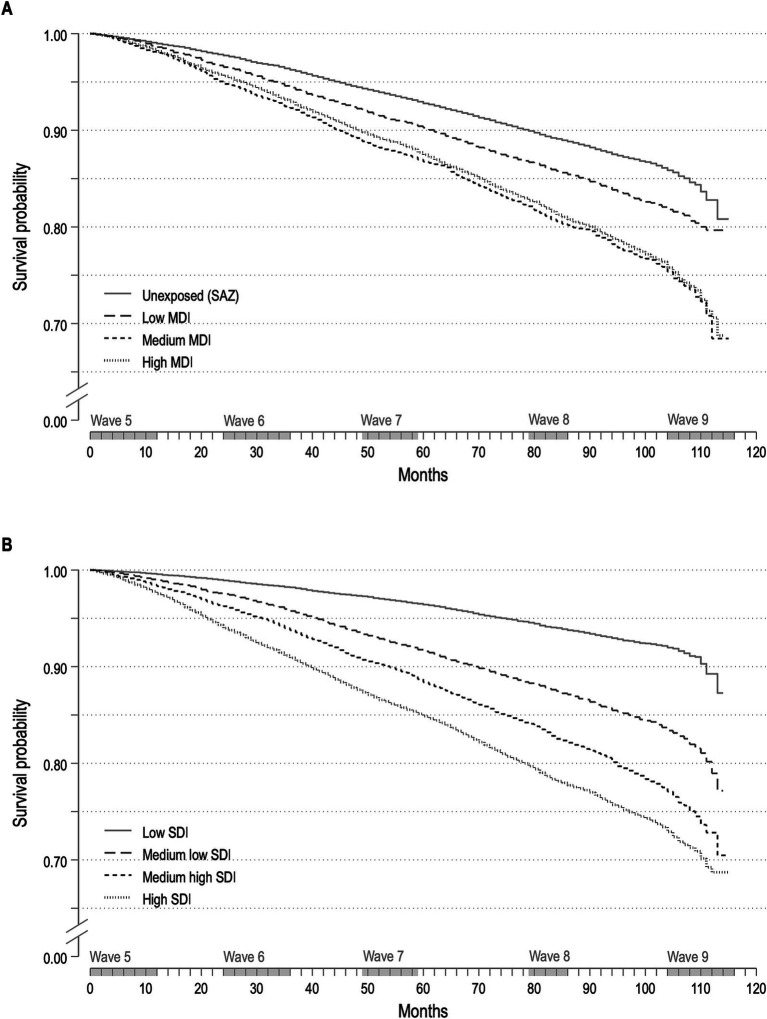
Kaplan–Meier estimates for material deprivation **(A)** and social deprivation **(B)** categories.

Figure 2 illustrates the all-cause survivor function for discrete subgroups of MDI (Figure 2A) and SDI (Figure 2B). Individuals who were not exposed to material deprivation (51.8%) and those with low MDI (19.0%) exhibited superior survival outcomes compared to those with medium and high MDI. A comparable pattern can be discerned regarding SDI, albeit with a more pronounced survival advantage from low to high SDI quartiles.

The results of the adjusted Cox regression between the Gini index and individual mortality (basic model) indicated a positive association between higher levels of regional income inequality and an increased risk of mortality from all causes (HR = 1.06, 95% CI = [1.02, 1.10], *R*^2^ = 0.738). However, no association was observed between the Gini index and the risk of mortality from cancer (HR = 1.03, 95% CI = [0.98, 1.07], *R*^2^ = 0.515), heart attack (HR = 1.05, 95% CI = [0.98, 1.13], *R*^2^ = 0.707) or stroke (HR = 1.00, 95% CI = [0.89, 1.07], *R*^2^ = 0.759).

**Figure 3:**
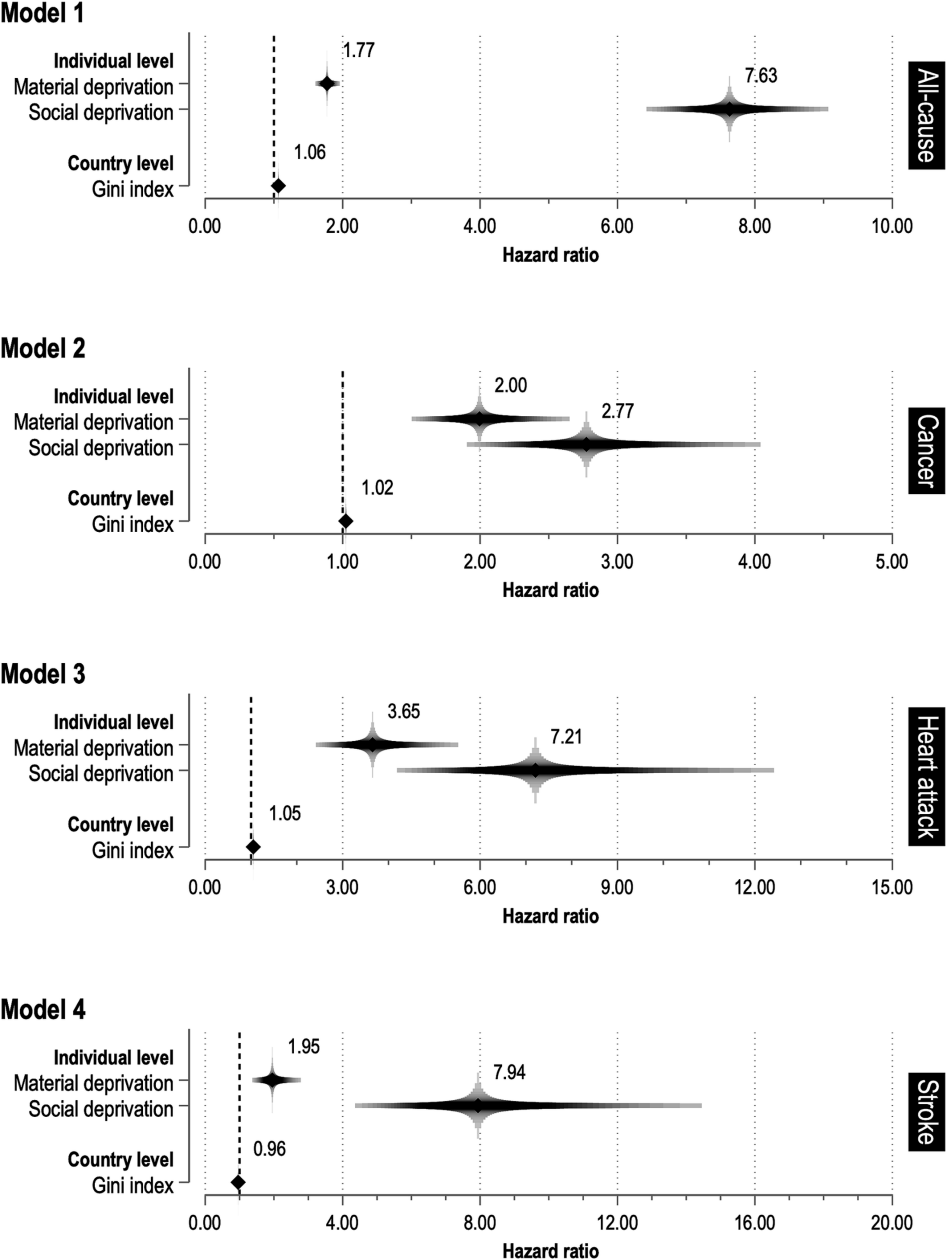
Relationship between socioeconomic deprivation and causes of death.

Figure 3 presents HRs and 95% CIs for the effects of individual-level socioeconomic deprivation and the Gini index on individual mortality risk (full model). The full model results, including estimates for covariates, are provided in Supplementary material S5. The results demonstrate that SDI exerts a larger effect on mortality in terms of HR. In Model 1 (*R*^2^ = 0.764), there was no longer evidence for a relationship between the Gini index and the individual all-cause mortality risk. However, the mortality risk for respondents with social deprivation was more than seven times that of respondents without social deprivation (HR = 7.63, 95% CI = [6.42, 9.07]). Similarly, respondents with material deprivation exhibited a higher risk of mortality than those without (HR = 1.77, 95% CI = [1.60, 1.96]). Similarly, Model 2 (*R*^2^ = 0.531) also revealed no relationship between the Gini index and the individual cancer mortality risk. Nevertheless, respondents with material and social deprivation exhibited a mortality risk that was more than twice that of respondents without material (HR = 2.00, 95% CI = [1.50, 2.65]) and social deprivation (HR = 2.77, 96% CI = [1.90, 4.04]), respectively. The results of Model 3 (*R*^2^ = 0.749) demonstrate that respondents with material and social deprivation exhibited a mortality risk due to heart attack that was more than three times and eight times higher, respectively, than that observed in individuals without material (HR = 3.65, 95% CI = [2.42, 5.52]) and social deprivation (HR = 7.21, 95% CI = [4.18, 12.41]). However, no evidence was found to indicate a relation between the Gini index and the individual risk of mortality from heart attacks. The results of Model 4 (*R*^2^ = 0.785) indicate that individuals with material deprivation exhibited a higher risk of stroke mortality in comparison to those without (HR = 1.95, 95% CI = [1.37, 2.77]). In contrast, the risk of stroke mortality for those with social deprivation was eight times that of individuals without (HR = 7.94, 95% CI = [4.36, 14.45]). As with the preceding disease-specific models, no evidence was found to indicate a relation between the Gini index and the individual risk of mortality from stroke.

Table 3 presents the results of the association between individual socioeconomic deprivation and individual mortality stratified by quintiles of the Gini index. No mortality-specific pattern was identified, nor was there any evidence to suggest a difference or trend for MDI and SDI through quintiles of the Gini index, as indicated by the overlap of the 95% CIs. However, it is notable that SDI shows the largest effects in the lower and middle quintiles (except for heart attack), while MDI shows the largest effects, especially for heart attack in regions with the lowest deprivation. Overall, the findings indicate that there is weak evidence to suggest an association between individual mortality from all causes, cancer, heart attack, or stroke, and the Gini index as a measure of area-level socioeconomic inequality. Conversely, there is substantial evidence to suggest an association between individual socioeconomic deprivation and individual mortality.

**Table 3:** Cox regression models with gamma shared frailty and random intercept for country of residence examining the association between material and social deprivation and cause of death, stratified by quintiles of the Gini index in the country of residence.

	All-cause^+^			Cancer^+^			Heart attack^+^			Stroke^+^		
	HR	95% CI		HR	95% CI		HR	95% CI		HR	95% CI	
		Lower	Upper		Lower	Upper		Lower	Upper		Lower	Upper
Q1, area of least inequality												
Material deprivation	2.14	1.74	2.64	2.17	1.19	4.21	8.21	3.37	19.96	1.88	0.98	3.61
Social deprivation	6.09	4.19	8.85	2.75	1.13	6.68	7.34	2.19	24.53	5.61	1.73	18.20
Q2												
Material deprivation	2.02	1.56	2.61	1.76	0.70	4.45	4.26	0.92	19.83	5.69	1.78	18.10
Social deprivation	15.98	9.94	25.70	5.15	1.82	14.53	2.29	0.30	17.63	16.29	1.95	135.58
Q3												
Material deprivation	1.44	1.07	1.93	1.11	0.43	2.85	3.63	0.81	16.25	1.35	0.46	3.97
Social deprivation	13.72	8.33	22.60	4.31	1.61	11.59	11.66	2.25	60.32	38.78	6.41	234.53
Q4												
Material deprivation	1.67	1.37	2.04	2.68	1.55	4.66	2.85	1.18	6.92	1.96	0.97	3.99
Social deprivation	5.24	3.74	7.34	2.52	1.16	5.47	3.72	1.09	12.63	6.29	2.01	19.67
Q5, area of greatest inequality												
Material deprivation	1.68	1.38	2.04	2.01	1.23	3.27	2.87	1.52	5.42	1.90	0.93	3.92
Social deprivation	7.79	5.59	10.86	2.26	1.14	4.48	12.89	5.48	30.32	6.67	1.99	22.37

HR, hazard ratio; CI, confidence interval + adjusted for age, sex, education, migration background, and Charlson comorbidity index.

## Discussion

This study examined the effect of individual and regional socioeconomic deprivation on individual mortality risk among older people in the European Region. The findings indicate that the Gini index, which served as a measure for regional deprivation, exerts a modest contextual effect on individual mortality risk for all causes and cancer, once sociodemographic characteristics are taken into account. However, the inclusion of individual-level deprivation in the models did not result in any discernible contextual effects. Instead, it became evident that individual socioeconomic status plays a more significant role in determining the actual causes of death among older individuals in the European Region, which is known as a compositional effect (43).

Previous multilevel studies that have examined the contextual effect of income inequality on mortality have yielded comparable results after adjusting for individual socioeconomic status (44, 45). However, the assertion that an individual’s socioeconomic status is the sole determinant of their mortality risk is not supported by the findings of this study. Prior research (46–49) also indicates that the individual effect may vary depending on contextual factors, underscoring the necessity for a more comprehensive study that incorporates these influences. In this study, the effect size of area-level deprivation may have been underestimated because socioeconomic heterogeneity within countries was not taken into account. This is because individual SHARE data were only available at the NUTS 0 level and in some cases at the NUTS 1 level for data protection reasons. Therefore, regional deprivation could only be considered at this aggregated level. It would be beneficial for future studies to attempt comparisons between spatial units at a more granular level, such as local administrative units within each country in the European Region. The regions under consideration exhibit considerable variation in population size and socioeconomic deprivation, with considerable heterogeneity within countries. When deprivation and mortality data are analyzed at a smaller scale, conclusions may change, a phenomenon known as the modifiable area unit problem (50). However, since these data are not available for all of Europe, small-scale analyses are very difficult to perform.

In contrast, the findings of this study indicate that individual socioeconomic deprivation, particularly in the domains of material and social resources, is associated with an increased risk of individual mortality from all causes, as well as specific conditions such as cancer, heart attack, and stroke. This finding is consistent with previous research from Europe (44, 51, 52), the United States (45, 53), and Asia (17), although individual socioeconomic status has typically been operationalized in terms of educational attainment, income, and occupation. A single prospective cohort study conducted in France was identified that examined the one-year survival rate following a stroke in patients with and without individual social deprivation (54). The findings of the French study indicated that patients with social deprivation exhibited a mortality risk that was more than twice that observed in patients without. However, the results of this study demonstrated that mortality rates were increased for both material deprivation and social deprivation, but with larger effects observed for social deprivation.

This study’s primary strength is its use of prospective data from the most comprehensive pan-European social science panel study (31), merged with area-level data obtained from the LIS Database (35). The study was limited by the exclusive use of a single indicator, the Gini index, for the investigation of regional socioeconomic deprivation. This was due to the unavailability of a validated multidimensional instrument for all European countries included in this study. For example, the European Deprivation Index is currently available for England, Italy, Portugal, Slovenia, and Spain (55, 56). It is thus imperative to recognize that the Gini index serves as a metric for income inequality, rather than a gauge for socioeconomic deprivation. The application of the Gini index is limited by the possibility that an area may exhibit a low Gini index due to its socially homogeneous nature, yet still demonstrate considerable socioeconomic deprivation in absolute terms or, conversely, display high levels of affluence. Consequently, statistical models were adjusted for the relative poverty rate. Moreover, a restricted number of social deprivation items, such as local vandalism and the accessibility of community resources, are indicative of characteristics intrinsic to the social context, situated at the meso level rather than the individual level. Nevertheless, the individual’s lifeworld is shaped by social behaviors and individual consumption patterns, including residential choices, among other factors. Additionally, the SHARE data set is constrained by the inclusion of self-reported diagnoses and a paucity of data concerning disease-specific obstacles and additional factors that may have influenced the vital status of the respondents.

Despite these limitations, this study contributes to our understanding of the relationship between socioeconomic deprivation and the underlying causes of mortality among older people in Europe. Further research is needed to clarify this relationship and to develop targeted health strategies at the European level. This will facilitate the reduction of social inequalities and their impact on health outcomes, thereby contributing to the promotion of social justice.

## Conclusion

The results confirm that individual socioeconomic status is a main predictor of actual causes of death among older adults in Europe. Individuals with material deprivation and social deprivation had a higher risk of mortality from all causes, as well as from cancer, heart attack, and stroke, compared to less disadvantaged individuals. The Gini index shows a negligible additional contextual effect on individual mortality, at least partly because of its inherent limitations as a measure of absolute deprivation. Nevertheless, the Gini index shows regional disparities across Europe, and future studies should examine regional measures of deprivation at a more granular level.

## Data Availability

Publicly available datasets were analyzed in this study. This data can be found here: the individual data sets analyzed during the current study are distributed by SHARE-ERIC to registered users through the SHARE Research Data Center. This paper uses data from SHARE Waves 5 (DOI: 10.6103/SHARE.w5.900), 6 (DOI: 10.6103/SHARE.w6.900), 7 (DOI: 10.6103/SHARE.w7.900), 8 (DOI: 10.6103/SHARE.w8.900), and 9 (DOI: 10.6103/SHARE.w9.900). More information about the data resource profile can be found here: Börsch-Supan et al. (31). The area-level data sets analyzed during the current study are distributed by the Luxembourg Income Study (LIS) Database, http://www.lisdatacenter.org (multiple countries; 2015–2021) Luxembourg: LIS.

## References

[1] World Health Organization. European mortality database. (2023). Available at: https://gateway.euro.who.int/en/datasets/european-mortality-database/ (Accessed July 2, 2024).

[2] Santucci C Carioli G Bertuccio P Malvezzi M Pastorino U Boffetta P. Progress in cancer mortality, incidence, and survival: a global overview. Eur J Cancer Prev. (2020) 29:367–81. doi: 10.1097/CEJ.0000000000000594 32740162

[3] GBD 2019 Stroke Collaborators. Global, regional, and national burden of stroke and its risk factors, 1990-2019: a systematic analysis for the Global Burden of Disease Study 2019. Lancet Neurol. (2021) 20:795–820. doi: 10.1016/S1474-4422(21)00252-0, PMID: 34487721 PMC8443449

[4] Aminorroaya A Yoosefi M Rezaei N Shabani M Mohammadi E Fattahi N. Global, regional, and national quality of care of ischaemic heart disease from 1990 to 2017: a systematic analysis for the global burden of disease study 2017. Eur J Prev Cardiol. (2022) 29:371–9. doi: 10.1093/eurjpc/zwab066, PMID: 34041535

[5] Roth GA Mensah GA Johnson CO Addolorato G Ammirati E Baddour LM. Global burden of cardiovascular diseases and risk factors, 1990-2019: update from the GBD 2019 study. J Am Coll Cardiol. (2020) 76:2982–3021. doi: 10.1016/j.jacc.2020.11.010, PMID: 33309175 PMC7755038

[6] Conrad N Molenberghs G Verbeke G Zaccardi F Lawson C Friday JM. Trends in cardiovascular disease incidence among 22 million people in the UK over 20 years: population based study. BMJ. (2024) 385:e078523. doi: 10.1136/bmj-2023-078523 38925788 PMC11203392

[7] Bidoli E Lamaj E Angelin T Forgiarini O De Santis E Serraino D. Linearity of age at cancer onset worldwide: 25-year population-based cancer registry study. Cancers. (2021) 13:5589. doi: 10.3390/cancers13215589, PMID: 34771751 PMC8583131

[8] Zahed H Feng X Sheikh M Bray F Ferlay J Ginsburg O. Age at diagnosis for lung, colon, breast and prostate cancers: an international comparative study. Int J Cancer. (2024) 154:28–40. doi: 10.1002/ijc.34671, PMID: 37615573 PMC11153845

[9] Townsend N Kazakiewicz D Lucy Wright F Timmis A Huculeci R Torbica A. Epidemiology of cardiovascular disease in Europe. Nat Rev Cardiol. (2022) 19:133–43. doi: 10.1038/s41569-021-00607-3 34497402

[10] Santos JV Padron-Monedero A Bikbov B Grad DA Plass D Mechili EA. The state of health in the European Union (EU-27) in 2019: a systematic analysis for the global burden of disease study 2019. BMC Public Health. (2024) 24:1374. doi: 10.1186/s12889-024-18529-3, PMID: 38778362 PMC11110444

[11] Vandenberghe D Albrecht J. The financial burden of non-communicable diseases in the European Union: a systematic review. Eur J Pub Health. (2020) 30:833–9. doi: 10.1093/eurpub/ckz073, PMID: 31220862

[12] Kölegård Stjärne M Diderichsen F Reuterwall C Hallqvist J. Socioeconomic context in area of living and risk of myocardial infarction: results from Stockholm heart epidemiology program (SHEEP). J Epidemiol Community Health. (2002) 56:29–35. doi: 10.1136/jech.56.1.29, PMID: 11801617 PMC1731992

[13] Macintyre K Stewart S Chalmers J Pell J Finlayson A Boyd J. Relation between socioeconomic deprivation and death from a first myocardial infarction in Scotland: population based analysis. BMJ. (2001) 322:1152–3. doi: 10.1136/bmj.322.7295.1152, PMID: 11348909 PMC31592

[14] Okui T Matoba T Nakashima N. The association between the socioeconomic deprivation level and ischemic heart disease mortality in Japan: an analysis using municipality-specific data. Epidemiol Health. (2022) 44:e2022059. doi: 10.4178/epih.e2022059, PMID: 35879856 PMC9754915

[15] Belau MH Becher H Riefflin M Bartig D Schwettmann L Schwarzbach CJ. The impact of regional deprivation on stroke incidence, treatment, and mortality in Germany. Neurol Res Pract. (2023) 5:6. doi: 10.1186/s42466-023-00232-0, PMID: 36755347 PMC9909858

[16] Marshall IJ Wang Y Crichton S McKevitt C Rudd AG Wolfe CD. The effects of socioeconomic status on stroke risk and outcomes. Lancet Neurol. (2015) 14:1206–18. doi: 10.1016/S1474-4422(15)00200-8 26581971

[17] Vathesatogkit P Batty GD Woodward M. Socioeconomic disadvantage and disease-specific mortality in Asia: systematic review with meta-analysis of population-based cohort studies. J Epidemiol Community Health. (2014) 68:375–83. doi: 10.1136/jech-2013-203053, PMID: 24407596

[18] Donkers H Bekkers R Galaal K. Systematic review on socioeconomic deprivation and cervical Cancer: inequalities in survival. J Health Care Poor Underserved. (2021) 32:751–66. doi: 10.1353/hpu.2021.0103, PMID: 34120975

[19] Finke I Behrens G Weisser L Brenner H Jansen L. Socioeconomic differences and lung cancer survival-systematic review and meta-analysis. Front Oncol. (2018) 8:536. doi: 10.3389/fonc.2018.00536 30542641 PMC6277796

[20] Green LW. Manual for scoring socioeconomic status for research on health behavior. Public Health Rep. (1896-1970) 85:815–27. doi: 10.2307/4593972 PMC2031767 4989476

[21] Adena M Myck M. Poverty and transitions in health in later life. Soc Sci Med. (2014) 116:202–10. doi: 10.1016/j.socscimed.2014.06.045, PMID: 25042393

[22] Pilehvari A You W Lin X. Retirement's impact on health: what role does social network play? Eur J Ageing. (2023) 20:14. doi: 10.1007/s10433-023-00759-w, PMID: 37162581 PMC10172431

[23] Kauppi M Virtanen M Pentti J Aalto V Kivimäki M Vahtera J. Social network ties before and after retirement: a cohort study. Eur J Ageing. (2021) 18:503–12. doi: 10.1007/s10433-021-00604-y, PMID: 34786012 PMC8563893

[24] Xue B Head J McMunn A. The impact of retirement on cardiovascular disease and its risk factors: a systematic review of longitudinal studies. Gerontologist. (2020) 60:e367–77. doi: 10.1093/geront/gnz062, PMID: 31091304 PMC7362617

[25] Li W Ye X Zhu D He P. The longitudinal association between retirement and depression: a systematic review and meta-analysis. Am J Epidemiol. (2021) 190:2220–30. doi: 10.1093/aje/kwab125, PMID: 33913467

[26] Silva IGP Marquete VF Lino IGT Batista VC Magnabosco G Haddad M. Factors associated with quality of life in retirement: a systematic review. Rev Bras Med Trab. (2022) 20:676–84. doi: 10.47626/1679-4435-2022-876, PMID: 37101432 PMC10124805

[27] Noble M Wright G Smith G Dibben C. Measuring multiple deprivation at the small-area level. Environ Plann A Econ Space. (2006) 38:169–85. doi: 10.1068/a37168

[28] Trinidad S Brokamp C Mor Huertas A Beck AF Riley CL Rasnik E. Use of area-based socioeconomic deprivation indices: a scoping review and qualitative analysis. Health Aff. (2022) 41:1804–11. doi: 10.1377/hlthaff.2022.00482, PMID: 36469826

[29] McNamara CL Balaj M Thomson KH Eikemo TA Solheim EF Bambra C. The socioeconomic distribution of non-communicable diseases in Europe: findings from the European social survey (2014) special module on the social determinants of health. Eur J Pub Health. (2017) 27:22–6. doi: 10.1093/eurpub/ckw222, PMID: 28355638

[30] Mackenbach JP Stirbu I Roskam AJ Schaap MM Menvielle G Leinsalu M. Socioeconomic inequalities in health in 22 European countries. N Engl J Med. (2008) 358:2468–81. doi: 10.1056/NEJMsa0707519, PMID: 18525043

[31] Börsch-Supan A Brandt M Hunkler C Kneip T Korbmacher J Malter F. Data resource profile: the survey of health, ageing and retirement in Europe (SHARE). Int J Epidemiol. (2013) 42:992–1001. doi: 10.1093/ije/dyt088, PMID: 23778574 PMC3780997

[32] Bertoni M Cavapozzi D Celidoni M Trevisan E. Development and validation of a material deprivation index In: Börsch-Supan A Kneip T Litwin H Myck M Weber G, editors. Ageing in Europe - supporting policies for an inclusive society. Berlin: De Gruyter (2015). 57–65.

[33] Hu Y van Lenthe FJ Mackenbach JP. Income inequality, life expectancy and cause-specific mortality in 43 European countries, 1987-2008: a fixed effects study. Eur J Epidemiol. (2015) 30:615–25. doi: 10.1007/s10654-015-0066-x, PMID: 26177800 PMC4579249

[34] Kõlves K Milner A Värnik P. Suicide rates and socioeconomic factors in eastern European countries after the collapse of the Soviet Union: trends between 1990 and 2008. Sociol Health Illn. (2013) 35:956–70. doi: 10.1111/1467-9566.12011 23398609

[35] Luxembourg income study (LIS) database. Available at: http://www.lisdatacenter.org (multiple countries; 2013-2021). Luxembourg: LIS.

[36] Bertoni M Cavapozzi D Celidoni M Trevisan E. Assessing the material deprivation of older Europeans In: Börsch-Supan A Kneip T Litwin H Myck M Weber G, editors. Ageing in Europe - supporting policies for an inclusive society. Berlin: De Gruyter (2015). 49–56.

[37] Adena M Myck M Oczkowska M. Material deprivation items in SHARE wave 5 data: a contribution to a better understanding of differences in material conditions in later life In: Börsch-Supan A Kneip T Litwin H Myck M Weber G, editors. Ageing in Europe - supporting policies for an inclusive society. Berlin: De Gruyter (2015). 25–37.

[38] Myck M Najsztub M Oczkowska M. Measuring social deprivation and social exclusion In: Börsch-Supan A Kneip T Litwin H Myck M Weber G, editors. Ageing in Europe - supporting policies for an inclusive society. Berlin: De Gruyter (2015). 67–77.

[39] Charlson ME Pompei P Ales KL MacKenzie CR. A new method of classifying prognostic comorbidity in longitudinal studies: development and validation. J Chronic Dis. (1987) 40:373–83. doi: 10.1016/0021-9681(87)90171-8, PMID: 3558716

[40] Quan H Li B Couris CM Fushimi K Graham P Hider P. Updating and validating the Charlson comorbidity index and score for risk adjustment in hospital discharge abstracts using data from 6 countries. Am J Epidemiol. (2011) 173:676–82. doi: 10.1093/aje/kwq433, PMID: 21330339

[41] Royston P. Explained variation for survival models. Stata J. (2006) 6:83–96. doi: 10.1177/1536867X0600600105

[42] Lorenz E Jenkner C Sauerbrei W Becher H. Modeling variables with a spike at zero: examples and practical recommendations. Am J Epidemiol. (2017) 185:650–60. doi: 10.1093/aje/kww122, PMID: 28369154

[43] Kawachi I Subramanian SV Almeida-Filho N. A glossary for health inequalities. J Epidemiol Community Health. (2002) 56:647–52. doi: 10.1136/jech.56.9.647, PMID: 12177079 PMC1732240

[44] Ng N Lundevaller E Malmberg G Edvinsson S. Income inequality and old-age mortality in Sweden: do regional development and lagged effect matter? Health Place. (2020) 64:102384. doi: 10.1016/j.healthplace.2020.102384, PMID: 32838898

[45] Fiscella K Franks P. Poverty or income inequality as predictor of mortality: longitudinal cohort study. BMJ. (1997) 314:1724–7. doi: 10.1136/bmj.314.7096.1724, PMID: 9185498 PMC2126895

[46] Lochner K Pamuk E Makuc D Kennedy BP Kawachi I. State-level income inequality and individual mortality risk: a prospective, multilevel study. Am J Public Health. (2001) 91:385–91. doi: 10.2105/AJPH.91.3.385, PMID: 11236402 PMC1446602

[47] Daly MC Duncan GJ Kaplan GA Lynch JW. Macro-to-micro links in the relation between income inequality and mortality. Milbank Q. (1998) 76:315. PMID: 9738166 10.1111/1468-0009.00094 PMC2751088

[48] Shin J Choi Y Kim SW Lee SG Park EC. Cross-level interaction between individual socioeconomic status and regional deprivation on overall survival after onset of ischemic stroke: national health insurance cohort sample data from 2002 to 2013. J Epidemiol. (2017) 27:381–8. doi: 10.1016/j.je.2016.08.020, PMID: 28688749 PMC5549246

[49] Reiss K Berger U Winkler V Voigtländer S Becher H Razum O. Assessing the effect of regional deprivation on mortality avoiding compositional bias: a natural experiment. J Epidemiol Community Health. (2013) 67:213–8. doi: 10.1136/jech-2012-201336 23093522

[50] Schuurman N Bell N Dunn JR Oliver L. Deprivation indices, population health and geography: an evaluation of the spatial effectiveness of indices at multiple scales. J Urban Health. (2007) 84:591–603. doi: 10.1007/s11524-007-9193-3, PMID: 17447145 PMC2219571

[51] Huisman M Kunst AE Bopp M Borgan JK Borrell C Costa G. Educational inequalities in cause-specific mortality in middle-aged and older men and women in eight western European populations. Lancet. (2005) 365:493–500. doi: 10.1016/S0140-6736(05)17867-2, PMID: 15705459

[52] Gallo V Mackenbach JP Ezzati M Menvielle G Kunst AE Rohrmann S. Social inequalities and mortality in Europe--results from a large multi-national cohort. PLoS One. (2012) 7:e39013. doi: 10.1371/journal.pone.0039013, PMID: 22848347 PMC3405077

[53] Steenland K Henley J Thun M. All-cause and cause-specific death rates by educational status for two million people in two American Cancer Society cohorts, 1959-1996. Am J Epidemiol. (2002) 156:11–21. doi: 10.1093/aje/kwf001, PMID: 12076884

[54] Béjot Y Bourredjem A Mimeau E Joux J Lannuzel A Misslin-Tritsch C. Social deprivation and 1-year survival after stroke: a prospective cohort study. Eur J Neurol. (2021) 28:800–8. doi: 10.1111/ene.14614, PMID: 33098727

[55] Guillaume E Pornet C Dejardin O Launay L Lillini R Vercelli M. Development of a cross-cultural deprivation index in five European countries. J Epidemiol Community Health. (2016) 70:493–9. doi: 10.1136/jech-2015-205729, PMID: 26659762 PMC4853548

[56] Zadnik V Guillaume E Lokar K Žagar T Primic Žakelj M Launoy G. Slovenian version of the European deprivation index at municipal level. Zdr Varst. (2018) 57:47–54. doi: 10.2478/sjph-2018-0007, PMID: 29651315 PMC5894458

