# Supplementary material for: Material and social deprivation associated with public health actual causes of death among older people in Europe: longitudinal and multilevel results from the Survey of Health, Ageing and Retirement in Europe (SHARE)
Source: Front Public Health. 2024 Oct 29;12:1469203. doi: 10.3389/fpubh.2024.1469203 (PMC11556392; doi:10.3389/fpubh.2024.1469203)
Supplement: Supplementary file 1 [file Table_1.DOCX]

**Supplementary Material 1**

**Table S1:** Results of Cox regression modeling of the functional form of material deprivation with a spike at zero (SAZ)

with all-cause mortality as the dependent variable

| **Model** | **Method** | **Deviance** | **Deviance from Null model** | **Deviance difference from best FP2/First stage model** | **df** | ***P*-value^a^** |
| --- | --- | --- | --- | --- | --- | --- |
| 0 | Null model^b^ | 135448.32 | 0 |  |  |  |
| 1 | Linear | 135333.70 | 114.62 |  | 1 | <0.001 |
| 2 | Standard FP |  |  |  |  |  |
| 2a | Linear (default) | 135333.70 |  | 12.89 | 1 | <0.001 |
| 2b | FP1^c^ | 135321.49 |  | 0.68 | 2 | 0.712 |
| 2c | FP2 | 135320.81 | 61.38 |  | 4 | <0.001 |
| 3 | Linear + z | 135323.56 | 124.76 |  | 2 | <0.001 |
| 4 | FP-spike |  |  |  |  |  |
|  | First stage |  |  |  |  |  |
| 4a | Linear + z (default) | 135323.56 |  | 3.16 | 2 | 0.206 |
| 4b | FP1 + z^c^ | 135320.93 |  | 0.52 | 3 | 0.915 |
| 4c | FP2 + z | 135320.40 | 127.92 |  | 5 | <0.001 |
|  | Second stage |  |  |  |  |  |
| 4d | Linear (dropping z)^c^ | 135333.70 |  | 13.30 | 1 | <0.001 |
|  | z (dropping linear) | 135363.56 |  | 39.99 | 1 | <0.001 |
| FP, fractional polynomial; FP1, first-degree fractional polynomial; FP2, second-degree fractional polynomial; df, degrees of freedom; ^a^ *P* value for the given deviance difference from the previous columns; ^b^ Deviance of the model without material deprivation but including all covariates; ^c^ results from application of the function selection procedures “standard FP” and “FP-spike” | | | | | | |
| **Interpretation:** Model with FP1 function showed best fit | | | | | | |

**Table S2:** Results of Cox regression modeling of the functional form of material deprivation with a spike at zero (SAZ)

with cancer mortality as the dependent variable

| **Model** | **Method** | **Deviance** | **Deviance from Null model** | **Deviance difference from best FP2/First stage model** | **df** | ***P*-value^a^** |
| --- | --- | --- | --- | --- | --- | --- |
| 0 | Null model^b^ | 33810.16 | 0 |  |  |  |
| 1 | Linear | 33788.32 | 21.84 |  | 1 | <0.001 |
| 2 | Standard FP |  |  |  |  |  |
| 2a | Linear (default) | 33788.32 |  | 2.08 | 1 | 0.149 |
| 2b | FP1^c^ | 33788.32 |  | 2.08 | 2 | 0.149 |
| 2c | FP2 | 33786.24 | 23.92 |  | 4 | <0.001 |
| 3 | Linear + z | 33788.32 | 21.84 |  | 2 | <0.001 |
| 4 | FP-spike |  |  |  |  |  |
|  | First stage |  |  |  |  |  |
| 4a | Linear + z (default) | 33788.32 |  | 2.67 | 2 | 0.263 |
| 4b | FP1 + z^c^ | 33787.18 |  | 1.53 | 3 | 0.675 |
| 4c | FP2 + z | 33785.65 | 24.51 |  | 5 | <0.001 |
|  | Second stage |  |  |  |  |  |
| 4d | Linear (dropping z)^c^ | 33788.32 |  | 2.67 | 1 | 0.102 |
|  | z (dropping linear) | 33801.62 |  | 15.97 | 1 | <0.001 |
| FP, fractional polynomial; FP1, first-degree fractional polynomial; FP2, second-degree fractional polynomial; df, degrees of freedom; ^a^ *P* value for the given deviance difference from the previous columns; ^b^ Deviance of the model without material deprivation but including all covariates; ^c^ results from application of the function selection procedures “standard FP” and “FP-spike” | | | | | | |
| **Interpretation:** Model with linear function showed best fit) | | | | | | |

**Table S3:** Results of Cox regression modeling of the functional form of material deprivation with a spike at zero (SAZ)

with heart attack mortality as the dependent variable

| **Model** | **Method** | **Deviance** | **Deviance from Null model** | **Deviance difference from best FP2/First stage model** | **df** | ***P*-value^a^** |
| --- | --- | --- | --- | --- | --- | --- |
| 0 | Null model^b^ | 13267.40 | 0 |  |  |  |
| 1 | Linear | 13231.93 | 35.47 |  | 1 | <0.001 |
| 2 | Standard FP |  |  |  |  |  |
| 2a | Linear (default) | 13231.93 |  | 1.73 | 1 | 0.188 |
| 2b | FP1^c^ | 13231.52 |  | 1.32 | 2 | 0.517 |
| 2c | FP2 | 13230.20 | 37.19 |  | 4 | <0.001 |
| 3 | Linear + z | 13230.20 | 37.20 |  | 2 | <0.001 |
| 4 | FP-spike |  |  |  |  |  |
|  | First stage |  |  |  |  |  |
| 4a | Linear + z (default) | 13230.20 |  | 0.04 | 2 | 0.980 |
| 4b | FP1 + z^c^ | 13230.20 |  | 0.04 | 3 | 0.998 |
| 4c | FP2 + z | 13230.16 | 37.24 |  | 5 | <0.001 |
|  | Second stage |  |  |  |  |  |
| 4d | Linear (dropping z)^c^ | 13231.93 |  | 1.77 | 1 | 0.183 |
|  | z (dropping linear) | 13244.92 |  | 14.76 | 1 | <0.001 |
| FP, fractional polynomial; FP1, first-degree fractional polynomial; FP2, second-degree fractional polynomial; df, degrees of freedom; ^a^ *P* value for the given deviance difference from the previous columns; ^b^ Deviance of the model without material deprivation but including all covariates; ^c^ results from application of the function selection procedures “standard FP” and “FP-spike” | | | | | | |
| **Interpretation:** Model with linear function showed best fit | | | | | | |

**Table S4:** Results of Cox regression modeling of the functional form of material deprivation with a spike at zero (SAZ)

with stroke mortality as the dependent variable

| **Model** | **Method** | **Deviance** | **Deviance from Null model** | **Deviance difference from best FP2/First stage model** | **df** | ***P*-value^a^** |
| --- | --- | --- | --- | --- | --- | --- |
| 0 | Null model^b^ | 10575.96 | 0 |  |  |  |
| 1 | Linear | 10567.67 | 8.29 |  | 1 | 0.004 |
| 2 | Standard FP |  |  |  |  |  |
| 2a | Linear (default) | 10567.67 |  | 6.11 | 1 | 0.013 |
| 2b | FP1^c^ | 10564.04 |  | 2.48 | 2 | 0.289 |
| 2c | FP2 | 10561.56 | 14.40 |  | 4 | 0.006 |
| 3 | Linear + z | 10564.21 | 11.75 |  | 2 | 0.028 |
| 4 | FP-spike |  |  |  |  |  |
|  | First stage |  |  |  |  |  |
| 4a | Linear + z (default) | 10564.21 |  | 3.21 | 2 | 0.201 |
| 4b | FP1 + z^c^ | 10563.21 |  | 2.21 | 3 | 0.530 |
| 4c | FP2 + z | 10561.00 | 14.96 |  | 5 | 0.011 |
|  | Second stage |  |  |  |  |  |
| 4d | Linear (dropping z)^c^ | 10567.67 |  | 6.67 | 1 | 0.010 |
|  | z (dropping linear) | 10565.53 |  | 4.53 | 1 | 0.033 |
| FP, fractional polynomial; FP1, first-degree fractional polynomial; FP2, second-degree fractional polynomial; df, degrees of freedom; ^a^ *P* value for the given deviance difference from the previous columns; ^b^ Deviance of the model without material deprivation but including all covariates; ^c^ results from application of the function selection procedures “standard FP” and “FP-spike” | | | | | | |
| **Interpretation:** Model with FP1 function showed best fit | | | | | | |
