# Supplementary material for: Material and social deprivation associated with public health actual causes of death among older people in Europe: longitudinal and multilevel results from the Survey of Health, Ageing and Retirement in Europe (SHARE)
Source: Front Public Health. 2024 Oct 29;12:1469203. doi: 10.3389/fpubh.2024.1469203 (PMC11556392; doi:10.3389/fpubh.2024.1469203)
Supplement: Supplementary file 2 [file Table_2.DOCX]

**Supplementary Material S5**

**Table B1:** Cox regression models with gamma shared frailty and random intercept for country of residence examining the association between individual-level and area-level socioeconomic deprivation and cause of death

|  |  | **All-cause^+^** | | |  | **Cancer^+^** | | |  | **Heart attack^+^** | | |  | **Stroke^+^** | | | |
| --- | --- | --- | --- | --- | --- | --- | --- | --- | --- | --- | --- | --- | --- | --- | --- | --- | --- |
|  |  | **HR** | **95% CI** | |  | **HR** | **95% CI** | |  | **HR** | **95% CI** | |  | **HR** | **95% CI** | | |
|  |  |  | **Lower** | **Upper** |  |  | **Lower** | **Upper** |  |  | **Lower** | **Upper** |  |  | **Lower** | **Upper** | |
| **Individual-level variables** |  |  |  |  |  |  |  |  |  |  |  |  |  |  |  |  | |
| Material deprivation |  | 1.77 | 1.60 | 1.96 |  | 2.00 | 1.50 | 2.65 |  | 3.65 | 2.42 | 5.52 |  | 1.95 | 1.37 | 2.77 | |
| Social deprivation |  | 7.63 | 6.42 | 9.07 |  | 2.77 | 1.90 | 4.04 |  | 7.21 | 4.18 | 12.41 |  | 7.94 | 4.36 | 14.45 | |
| Age |  | 1.10 | 1.09 | 1.11 |  | 1.06 | 1.05 | 1.07 |  | 1.09 | 1.08 | 1.10 |  | 1.11 | 1.10 | 1.12 | |
| Sex |  |  |  |  |  |  |  |  |  |  |  |  |  |  |  |  | |
| Male |  | 1.00 |  |  |  | 1.00 |  |  |  | 1.00 |  |  |  | 1.00 |  |  | |
| Female |  | 0.54 | 0.51 | 0.56 |  | 0.55 | 0.50 | 0.61 |  | 0.37 | 0.31 | 0.43 |  | 0.60 | 0.51 | 0.71 | |
| Education |  |  |  |  |  |  |  |  |  |  |  |  |  |  |  |  | |
| Low |  | 1.00 |  |  |  | 1.00 |  |  |  | 1.00 |  |  |  | 1.00 |  |  | |
| Medium |  | 0.86 | 0.81 | 0.91 |  | 0.93 | 0.83 | 1.05 |  | 0.88 | 0.73 | 1.06 |  | 0.74 | 0.60 | 0.90 | |
| High |  | 0.66 | 0.61 | 0.71 |  | 0.67 | 0.58 | 0.78 |  | 0.72 | 0.57 | 0.92 |  | 0.60 | 0.47 | 0.79 | |
| Migration background |  |  |  |  |  |  |  |  |  |  |  |  |  |  |  |  | |
| Absent |  | 1.00 |  |  |  | 1.00 |  |  |  | 1.00 |  |  |  | 1.00 |  |  | |
| One-sided |  | 1.02 | 0.91 | 1.13 |  | 1.11 | 0.90 | 1.37 |  | 1.00 | 0.69 | 1.46 |  | 0.77 | 0.50 | 1.19 | |
| Two-sided |  | 1.06 | 0.98 | 1.14 |  | 1.02 | 0.88 | 1.19 |  | 1.13 | 0.89 | 1.43 |  | 1.16 | 0.92 | 1.48 | |
| Charlson comorbidity index |  | 0.25 | 0.24 | 0.27 |  | 0.29 | 0.26 | 0.32 |  | 0.26 | 0.22 | 0.31 |  | 0.30 | 0.25 | 0.35 | |
| **Area-level variables** |  |  |  |  |  |  |  |  |  |  |  |  |  |  |  |  | |
| Gini index |  | 1.06 | 1.02 | 1.11 |  | 1.02 | 0.98 | 1.07 |  | 1.05 | 0.98 | 1.12 |  | 0.96 | 0.88 | 1.04 | |
| Relative poverty rate |  | 1.42 | 0.37 | 5.44 |  | 3.44 | 0.32 | 36.39 |  | 0.08 | 0.02 | 2.60 |  | 3.68 | 0.44 | 31.13 | |
| Migrant population rate |  | 0.97 | 0.96 | 0.98 |  | 0.99 | 0.98 | 1.00 |  | 0.98 | 0.96 | 1.00 |  | 1.00 | 0.98 | 1.02 | |
| HR, hazard ratio; CI, confidence interval | | | | | | | | | | | | | | | | |  |
