# Supplementary figures and images for: Material and social deprivation associated with public health actual causes of death among older people in Europe: longitudinal and multilevel results from the Survey of Health, Ageing and Retirement in Europe (SHARE)
Source: Front Public Health. 2024 Oct 29;12:1469203. doi: 10.3389/fpubh.2024.1469203 (PMC11556392; doi:10.3389/fpubh.2024.1469203)

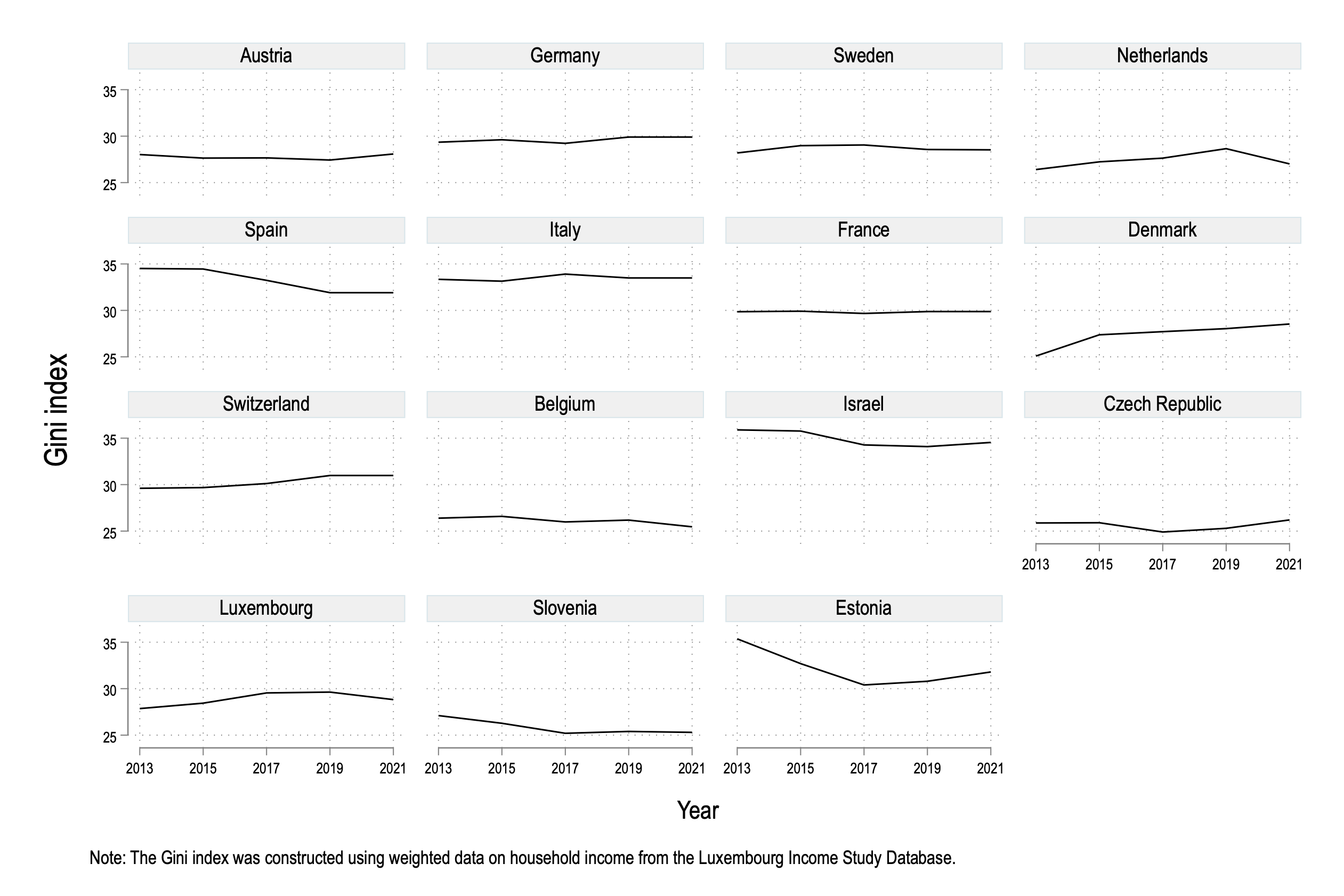

Supplement: Supplementary file 3 [file Image_1.TIF]

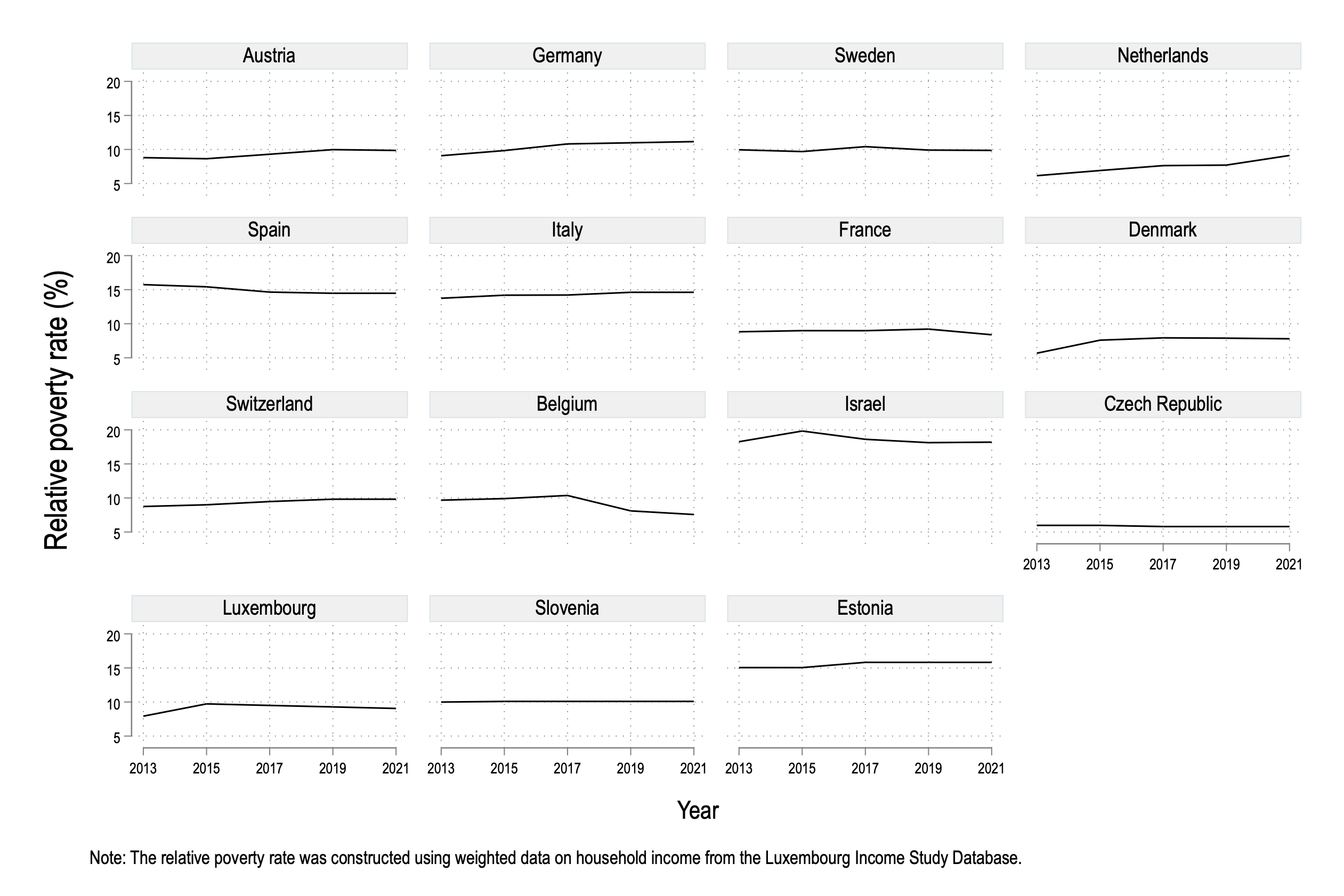

Supplement: Supplementary file 4 [file Image_2.TIF]

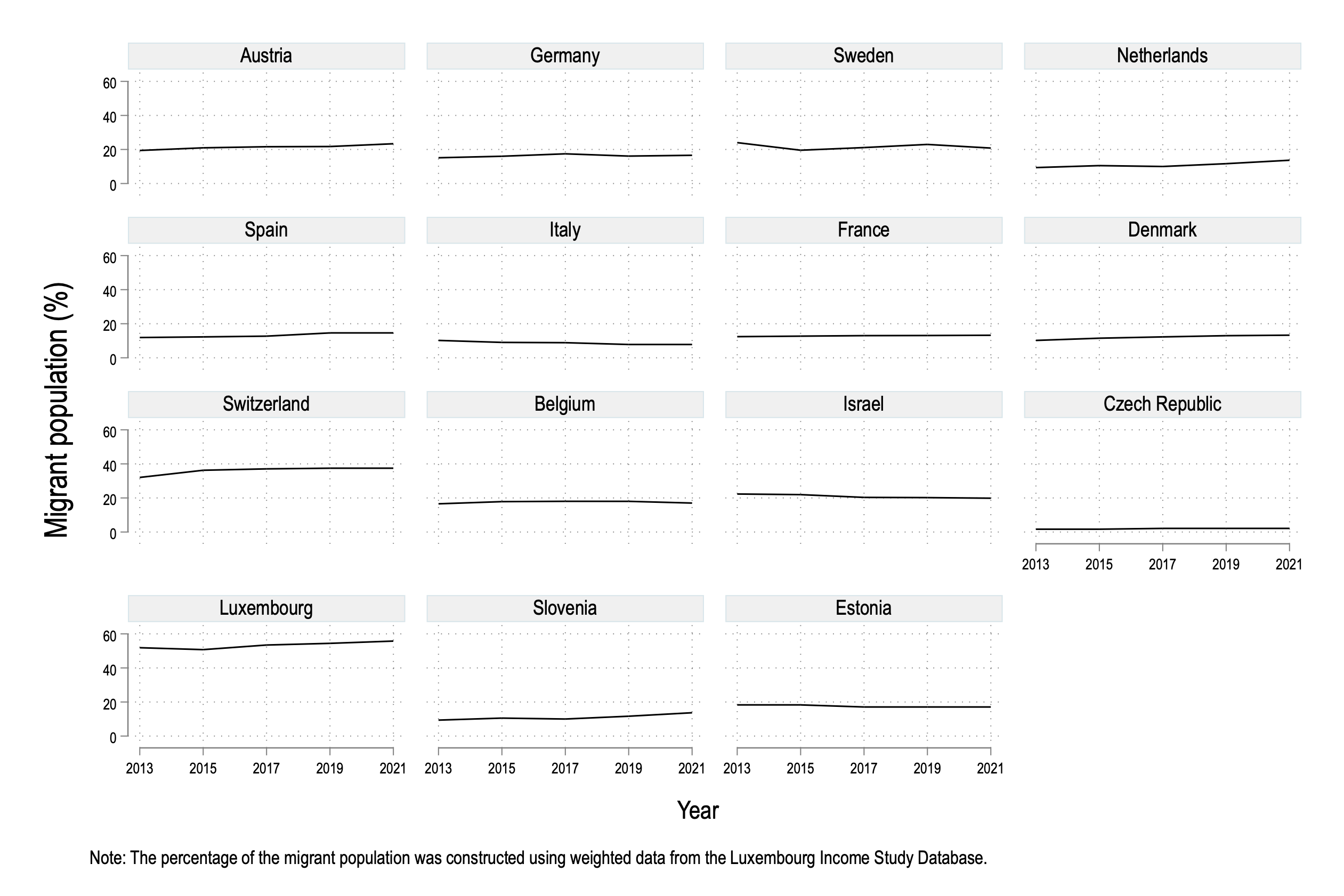

Supplement: Supplementary file 5 [file Image_3.TIF]
